# Viral Related Tools against SARS-CoV-2

**DOI:** 10.3390/v12101172

**Published:** 2020-10-16

**Authors:** Laura Fernandez-Garcia, Olga Pacios, Mónica González-Bardanca, Lucia Blasco, Inés Bleriot, Antón Ambroa, María López, German Bou, Maria Tomás

**Affiliations:** 1 Microbiology Department-Research Institute Biomedical A Coruña (INIBIC), Hospital A Coruña (CHUAC), University of A Coruña (UDC), 15006 A Coruña, Spain; laugemis@gmail.com (L.F.-G.); olgapacios776@gmail.com (O.P.); monica.gonzalez.bardanca@sergas.es (M.G.-B.); luciablasco@gmail.com (L.B.); bleriot.ines@gmail.com (I.B.); anton17@mundo-r.com (A.A.); maria.lopez.diaz@sergas.es (M.L.); German.Bou.Arevalo@sergas.es (G.B.); 2 Study Group on Mechanisms of Action and Resistance to Antimicrobials (GEMARA) of Spanish Society of Infectious Diseases and Clinical Microbiology (SEIMC), 28003 Madrid, Spain; 3 Spanish Network for the Research in Infectious Diseases (REIPI), 41071 Sevilla, Spain

**Keywords:** SARS-CoV-2, COVID-19, phages, CRISPR, viruses, prevention, diagnosis, treatment

## Abstract

At the end of 2019, a new disease appeared and spread all over the world, the COVID-19, produced by the coronavirus SARS-CoV-2. As a consequence of this worldwide health crisis, the scientific community began to redirect their knowledge and resources to fight against it. Here we summarize the recent research on viruses employed as therapy and diagnostic of COVID-19: (i) viral-vector vaccines both in clinical trials and pre-clinical phases; (ii) the use of bacteriophages to find antibodies specific to this virus and some studies of how to use the bacteriophages themselves as a treatment against viral diseases; and finally, (iii) the use of CRISPR-Cas technology both to obtain a fast precise diagnose of the patient and also the possible use of this technology as a cure.

## 1. Introduction

At the end of 2019, a new virus appeared in the city of Wuhan (China) and quickly spread throughout the world, causing a global pandemic. The virus is closely related to the SARS-CoV (Severe Acute Respiratory Syndrome Coronavirus), thus named SARS-CoV-2 [1,2]. It is a β-coronavirus, carrying single-stranded, positive-sense RNA genome and four main structural proteins: spike (with two subunits, S1 and S2), envelope, membrane and nucleocapsid (N) [3]. SARS-CoV-2, as SARS-CoV, enters the cell through the receptor-binding domain (RBD) of the S1, which recognizes the angiotensin-converting protein 2 (ACE2), present in the surface of host cells [4]. SARS-CoV-2 provokes COVID-19, a new disease that produces a wide range of symptoms ranging from an asymptomatic carrier state to respiratory distress syndrome and even acute heart injury with the risk of secondary infections [5]. The rapid spread of the virus and the absence of treatment for this new disease have led researchers all over the world to join forces in the search for a solution by using all available resources.

Since the beginning of this pandemic, all the medical resources were focused on two main points: diagnostic and treatment of the disease. The diagnosis of SARS-CoV-2 was firstly based on molecular approaches [6], the real-time RT-PCR assay has become the election method to detect the presence of the virus as it is a specific and sensitive method to disclose viral RNA from respiratory tract samples [7]. In order to establish the presence of the virus, following the WHO’s indications, clinical laboratories from all around the world are using various primer pairs: the spike gene, the RNA-dependent RNA polymerase gene (RdRp), the nucleocapsid gene and the envelope gene [8]. Besides, some serologic analyses have been used to diagnose an active or past infection by quantifying the presence of IgM and IgG in the patient serum [8]. An interesting systematic review and meta-analysis has been carried out concerning the serological assays [9]. Authors concluded that the sensitivity of this technique was higher three weeks after the symptom onset, compared with the first week, and that heterogeneity was found in all analyses. Among the advantages of serological assays, we find that they are cheaper and easier to implement at the point of care, but, above all, they can identify asymptomatic individuals previously infected by SARS-CoV-2. Moreover, serological tests could be deployed as surveillance tools to better understand the epidemiology of SARS-CoV-2. Many serological tests for Covid-19 have become available in a very short period, and this is precisely where their main disadvantage resides: the pace of development of serological tests has been so fast that it has exceeded that of rigorous evaluation. Therefore, uncertainty about the accuracy of serological assays remains important [9].

Concerning the treatment of this disease, and due to its rapid development, finding an effective treatment against it was imperative. Thus, researchers and medical doctors began to test existing medicines and repurposing them as COVID-19 treatments, highlighting: (i) nucleoside analogs, as favipiravir (used for influenza virus, Ebola, chikungunya, yellow fever, enterovirus and norovirus treatment) [10,11], ribavirin (used for treating the respiratory syncytial virus, hepatitis C virus and also against SARS and MERS) [12], remdesivir (used for HIV treatment) [11,13] or galidesivir [14]; (ii) antiparasitics as chloroquine (used against malaria, with positive in vitro results against SARS, MERS, Ebola, HIV, Nipah and Hendra viruses, although no protection was found in vivo against these viruses) [15,16,17,18]; (iii) protease inhibitors (lopinavir and ritonavir used as HIV treatments) [19]; (iv) indole-derivate molecules as arbidol (used against hepatitis viruses) [20]; and finally, (v) convalescent plasma therapy from patients who recovered from the infection [21].

Paradoxically, an efficient prevention strategy to combat SARS-CoV-2 could come from different human viruses, e.g., in the form of a vaccine vector. A virus is known as an extremely small infective particle, which can only replicate inside a host. Since their discovery, they have been identified as the cause of a great number of diseases, but more recently, they have also been considered a solution for some of them [22]. Viruses can be genetically modified to express antigens of interest, turning them into efficient vectors that deliver immunogenic particles inside the human body [23]. The usefulness of the viral vectors is based on: (i) their high specificity for their targets, (ii) their ability for gene transduction and (iii) their capacity to generate strong cellular and humoral immune responses without an adjuvant [22]. Besides, all the viruses used as vectors are genetically modified to eliminate their replicative capacity and to decrease or eradicate their pathogenicity. However, a potential problem with viral vectors is the pre-existing immunity, due to previous viral exposure [22].

Indeed, there are viruses able to specifically infect bacteria as well. These are called bacteriophages, and they can also represent an interesting tool useful in the analysis of SARS-CoV-2, in the diagnostic of the disease and in its treatment. Bacteriophages (also known as “phages”) are the natural predators of bacteria, highly specific: They recognize the bacterial receptors on the surface of the prokaryotic cells and strongly attach to them [24]. Since the discovery of bacteriophages in 1915 [25], they have been used as an alternative treatment for critical bacterial infections, on some occasions even life-threatening [26,27]. In the last decade, i.e., in the post-antibiotic era, the therapy based on lytic phages (phage therapy) or phage derived proteins (enzybiotics) such as, for instance, phage-encoded endolysins [28], has gained popularity, being one of the few options currently available for infections caused by multi-drug resistant (MDR) bacteria [26,29]. Phages have demonstrated their innocuousness for humans, although some concerns still need to be investigated such as the purity of the preparation [26]. However, this is far from being the only use for phages; they might be a good option to isolate neutralizing antibodies against other infectious diseases, caused by parasites [30] or viruses [31], using the phage display technique.

Highly related to bacteriophages are the Cluster Regulatory Interspaced Palindromic Repeats (CRISPR), discovered in 1993 and firstly named as short regulatory repeats (SRSRs) [32]. It was years later when their function as a bacterial immunity system against bacteriophages was reported [33,34]. CRISPR fragments are phage-derived sequences harbored by bacteria in their chromosomes that act as an acquired immunity system in prokaryotes: when a bacterium that has been infected by a bacteriophage is re-infected by the same type of phage, CRISPR-Cas system recognizes the viral DNA/RNA repeated sequences and digests the spacer segments between the repetitions, using the endonuclease activity of Cas (CRISPR associated) proteins [35]. This system has been extensively studied by many scientific researchers from all over the world and belonging to very different domains [36]. The importance of this technology has been increasing in the last decade, and nowadays, it is even possible to replace one DNA fragment by another; therefore, CRISPR-Cas system is currently considered one of the most important tools to genetic edition, treatment of diseases and genetic modification of mammalian cells, among others [36].

Throughout this work, we have analyzed innovative methods of diagnostic and treatment of this new disease, the COVID-19, based on the use of human viruses, bacterial viruses (bacteriophages), or virus-related tools (CRISPR). Due to the novelty of the topic here discussed and the amount of information available, in this review, some articles that have not been peer-reviewed are cited.

## 2. Human Viruses as Prevention

Nowadays, there are several types of viral vectors depending on the type of virus used: retrovirus [37], lentivirus [38], Sendai virus [39], cytomegalovirus [40], poxvirus [41], adenovirus [42], adeno-associated virus (AAV) [43], among others. These vectors have been used against several diseases such as HIV [44,45,46,47,48], hepatitis [49], tuberculosis [50,51], influenza [52,53] and even cancer [54,55]. The most common viruses used for the development of vaccines against human infectious diseases are poxvirus and adenovirus. Poxviruses were the first viruses ever used as vaccine and so the best known with a safety and efficacy widely demonstrated; on the other hand, adenoviruses have been deeply analyzed especially due to its easy production, great transduction efficiency, a broad spectrum of tropism and their transgene expression [22]. The following studies and/or clinical trials measured an elicited humoral response (quantified by ELISA or Western blot) and a neutralizing response (by neutralization assays using either the live virus or a pseudovirus). Neutralizing antibodies can, as their name implies, neutralize the biological effects of the antigen and interfere with their infectivity without a need for immune cells. Currently developed SARS-CoV- and MERS-CoV-specific neutralizing antibodies include monoclonal antibodies (mAbs), their functional antigen-binding fragment (Fab), the single-chain variable region fragment (scFv), or single-domain antibodies. They target S1-RBD, S1-NTD, or the S2 region, blocking the binding of RBDs to their respective receptors and interfering with S2-mediated membrane fusion or entry into the host cell, thus inhibiting viral infections [56].

One of the best-known poxvirus vectors is the Modified Vaccinia Ankara (MVA), unable to replicate in most mammalian cells, thus becoming a safe vector that expresses antigens which elicit an immune response [57]. MVA has been recently modified by Chiuppesi et al. to co-express SARS-CoV-2 spike (S) and nucleocapsid (N) antigens with the aim of testing its immunogenicity and developing a candidate vaccine against COVID-19. In the study, the authors challenged several mice with two MVA vectors, sMVA-S, and sMVA-N vectors, expressing the S and N antigen, respectively. Both vectors were evaluated in a murine model by co-immunization at different doses, and they observed similar SARS-CoV-2 antigen-specific humoral and cellular immune responses in vaccine groups receiving sMVA-S and sMVA-N alone or in combination. Authors claimed that both vectors expressing the S and N antigens can stimulate potent SARS-CoV-2-specific humoral and cellular immune responses in mice, either expressed isolated or in combination. For neutralizing experiments, they used SARS-CoV-2 pseudovirus and detected neutralizing antibodies in all vaccine groups receiving the S antigen. The authors claimed that these neutralizing responses increased after the booster immunization [57].

The adenoviral vector most commonly used for clinical trials and experimental gene therapy applications is HAdV-C5, abbreviated as Ad5 [58]. The research group of Zhu et al. performed a phase-2 trial using a replication-defective Ad5 expressing the spike glycoprotein of SARS-CoV-2, to assess its level of safety, tolerability, and immunogenicity in a group of healthy adults. This trial did not report serious adverse events within 28 days post-vaccination. They found a peak in specific T-cells at day 14 post-vaccination, whereas the peak in neutralizing antibodies anti-spike occurred at day 28 post-vaccination, detected through both live SARS-CoV-2 virus neutralization and pseudovirus neutralization tests [59].

Nowadays, this vaccine is being tested in humans in a phase-3 clinical trial (Table 1) [60]. Similarly, at the University of Oxford, scientists have designed a chimpanzee adenovirus (ChAdOx1) vectored vaccine encoding a codon-optimized full-length spike protein of SARS-CoV-2 [61]. The authors reported that a single vaccination with ChAdOx1 nCoV-19 was effective in preventing damage to the lungs upon high dose, indicating that vaccination prevents virus replication in the lower respiratory tract, but no reduction in viral shedding from the nose was observed. The biggest limitation of this study was that animals were challenged with a high dose of virus via multiple routes, which does not simulate a realistic human exposure [61]. These researchers performed a phase-1/2 randomized trial in healthy adults and observed that those vaccinated with the ChAdOx1 nCoV-19 (5 × 10^10^ viral particles) experimented a few mild/moderate secondary effects during the first days after the vaccination. Nevertheless, authors demonstrated that their vaccine is effective with a single-dose, without several adverse reactions, and detected the presence of high levels of neutralizing antibodies as well as spike-specific antibodies 28 days after vaccination [62]. Currently, this vaccine is in a phase-3 clinical trial in different countries (Table 1) [63].

Consistently with their preliminary results, Mercado et al. immunized several rhesus macaques with another adenoviral vector (Ad26) expressing also the spike protein. However, the immunogen that they used was the full-length membrane-bound S protein with a mutation of the furin cleavage site and two proline stabilizing mutations [64]. They reported a robust immune response based on neutralizing antibodies, obtaining complete protection against the SARS-CoV-2 challenge in 5 out of 6 animals [65]. Based on the previous results, they are now performing a phase-1/2 trial in healthy adults in which they are going to administrate two intramuscular doses of the vaccine (Ad26COVS1) [65,66].

In the same context, the Gamaleya Research Institute of Russia had performed two phase-1 clinical trials with adeno-based vaccines. In these clinical trials, they are going to test the safety of two different vaccines, one based in Ad26 and the other in Ad5, both containing the Spike protein of the SARS-CoV-2 and the lyophilizate of the two mentioned above, for the preparation of a solution for intramuscular injection [67,68]. The combination of Ad26 and Ad5 expressing the spike protein is now in phase-3 [67,68].

Moreover, companies from Italy, Germany, and Belgium have joined forces to develop a simian adenoviral vector-based vaccine that expressed the S protein of the SARS-CoV-2, whose phase 1 clinical trial has begun in Italy this summer [69].

Moreover, the Pasteur Institute in collaboration with two companies and the University of Pittsburgh have developed a Measles-vector vaccine expressing a modified surface glycoprotein of the SARS-CoV-2. This vaccine candidate is nowadays in phase 1 clinical trial, to test the safety, tolerability and immunogenicity of a vaccine that is going to be administrated intramuscularly in two doses separated by 28 days in 90 healthy adults [70]. Furthermore, Medicago Inc. has developed another phase-1 trial testing a virus-like particle vaccine that will be injected into healthy adults with or without an adjuvant, trying different doses of the vaccine [71].

Xiamen University is developing an intranasal spray viral-vector vaccine, based on influenza A virus expressing the spike protein (Table 1). This is currently in a phase-1 clinical trial, and the spray is being nasally administered in one dose in 60 healthy adults [72].

Apart from all these clinical trials, according to the World Health Organization (WHO), nowadays there are 49 viral-vector candidates in pre-clinical evaluation: 9 using adenoviruses, 4 using MVA, 7 using influenza A virus, 3 using Measles virus, 5 using VSV (vesicular stomatitis virus), 7 using other viruses, and 12 using virus-like particles [73] (Table 1).

## 3. Bacteriophages

**Figure 1:**
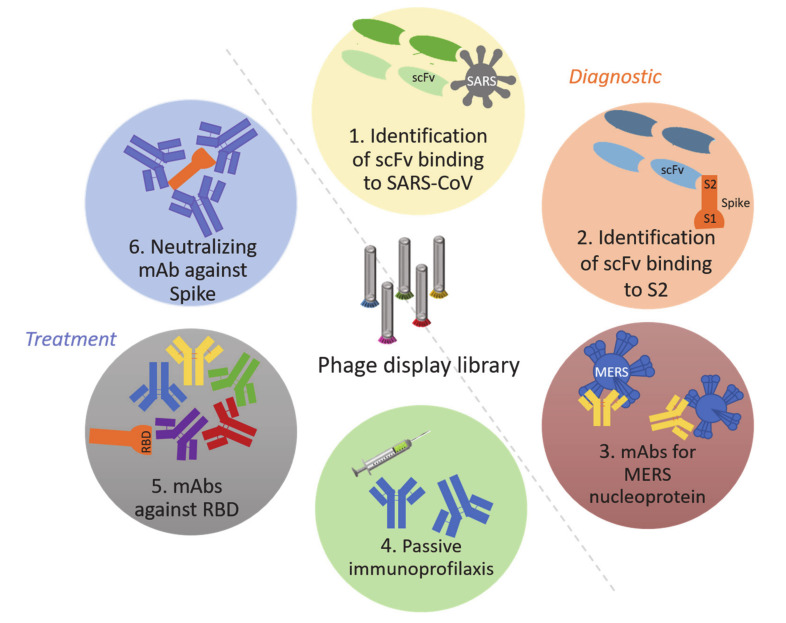
Uses of phage-display libraries in the diagnostic (1, 2 y 3) and treatment (4, 5, and 6) of SARS-CoV-2. scFv: single-chain variable fragment. S2: spike subunit 2. mAbs: monoclonal antibodies. MERS: Middle-East respiratory syndrome. RBD: receptor-binding protein.

As detailed above, bacteriophages are the natural viruses of bacteria that have been used to treat diseases for a long time. Therefore, in 1988, de la Cruz et al. modified the filamentous phage F1 from *Escherichia coli* in order to express repetitive regions from the circumsporozoite protein of *Plasmodium falciparum* [74]. The resulting phages displayed the recombinant protein on their capsid surface and were found to act as carriers capable of producing immunological responses in rabbits. This is one example of how one of the oldest and most abundant entities on Earth has been turned into a powerful therapeutic weapon. Since then, researchers have been investigating the potential of phages in the fight against other infectious diseases. Phage display libraries are a remarkably useful tool that allows the identification of the best ligands for a given target [75], permitting the construction of large libraries consisting of numerous antibody genes [76]. This type of libraries has been used since 1992 to identify specific monoclonal antibodies (mAb) against certain bacteria or viruses [77,78], and a high percentage of human therapeutic antibodies have been developed by this technique [79]. In the past, there have been several examples of phage display libraries expressing viral peptides that have successfully inhibited infections, for instance, the ones caused by adenovirus type 2 [80], hepatitis B virus [81], hantavirus, sin nombre virus [82], and Andes virus [83]. This justifies the use of these libraries as a diagnostic and treatment tools of SARS-CoV-2 (Figure 1).

### 3.1. Bacteriophages as Diagnostic Tools: Phage-Display Libraries

Phage display is a powerful technique for the identification and isolation of peptides or proteins [76]. This technique consists of expressing foreign peptides on the surface of bacteriophages, frequently filamentous bacteriophages isolated from *E. coli*, but not exclusively. Indeed, phagemids are the most commonly used vector in phage display technique: these filamentous-phage-derived vectors contain the replication origin of a plasmid, a selective marker, the intergenic region (usually containing the packing sequence), a gene of a phage coat protein, restriction enzyme recognition sites, a promoter and a DNA segment encoding a signal peptide [84]. Phagemids have small genomes, which makes them suitable to accommodate larger foreign DNA fragments. Moreover, they are more efficient in transformation, which allows for obtaining a phage display library with high diversity. A variety of restriction enzyme recognition sites are available in the genome of phagemids, which is convenient for DNA recombination and gene manipulation. Furthermore, the expression level of fusion proteins can be easily controlled and, finally, phagemids are usually genetically more stable than recombinant phages [84].

Nowadays, the most common phages used are the M13 (*Inoviridae*), T4 (*Myoviridae*), T7 (*Podoviridae*) and Lambda (*Syphoviridae*) [85]. Most of the proteins are displayed as fusion proteins with the N- or C-terminus of different phage surface proteins: coat proteins pIII or pVIII on M13 [86], capsid proteins HOC (highly antigenic outer capsid) or SOC (small outer capsid) on T4 [87], the capsid protein pX on T7 [88] and the head protein pD or the tail protein pV on Lambda phage [89]. To select a specific mAb from the library, phages must be subjected to a multiple-cycle process, and after each one, antibodies showing the highest affinity are chosen for the next round. During each cycle, the library is incubated with the target, previously immobilized on a solid support, washed, eluted, amplified and reselected [90]. In the case of phage display libraries expressing mAbs, these are often quantified by ELISA or similar techniques [91]. In the context of the current COVID-19 pandemic, it is worthy to consider that the use of this method has led to major discoveries concerning highly related coronavirus, like SARS or MERS: (i) identification of two single-chain fragment variable (scFv) antibodies that are highly specific for SARS-CoV [92] and (ii) proposal of a new method, called “Yin-Yang”, for selecting mAbs by using crude antigens of MERS-CoV (Middle East Respiratory Syndrome Coronavirus), which led to the isolation of three mAb against the MERS-CoV nucleocapsid protein [93]. Besides, several researchers have suggested highly specific diagnostic methods that use phage display libraries for the S1 subunit of the SARS-CoV spike protein, with no cross-reaction with other coronaviruses [75].

Phage display libraries have led to several discoveries associated with coronaviruses that have caused serious human diseases in the past, such as SARS-CoV or MERS. One example is the identification of an scFv antibody, called B1, which binds the S2 of the spike protein of the SARS-CoV both in vitro and in vivo, exhibiting a potent neutralizing activity [94]. Moreover, this same technique allowed for the detection of a human Fab (Fragment antigen-binding) molecule against the spike protein of SARS-CoV, named M1A that could be used in passive immunoprophylaxis [95]. However, the Fc (Fragment crystallizable) region of the antibody is needed to enable the development of a proper immune response [96], so structural modifications (as the authors suggested) would be interesting in order to enhance its protective and neutralizing capacities. Following with these libraries, five types of mAb against the receptor-binding domain (RBD) of the SARS-CoV-2 spike protein have been identified [97]. Finally, phage display libraries lead to the identification of two important mAbs, one of which neutralizes the RBD of the SARS-CoV-2 [98] (Figure 1).

### 3.2. Bacteriophages as Treatments

In addition to the phage-libraries, other strategies employing phages have been developed as treatments. Regarding the coronavirus type, Ren et al. developed phages that bear specific gastroenteritic coronavirus peptides, which induced humoral and cell-mediated immunity in mice, suggesting that phage-based vaccines may be efficient heterologous antigens for initiating host humoral and cellular immune responses [99]. Furthermore, Lauster et al. modified the icosahedral capsid of Q-beta-phage (Qβ) to display sialic acid ligands that bind to the trimeric haemagglutinin (HA) of the influenza A virus (IAV). These researchers demonstrated that the Qβ-phage capsids can act as highly specific inhibitors of IAV, completely blocking its entry to cells by covering the whole envelope of the virus. However, this method is still undergoing preclinical development [100].

## 4. CRISPR-Cas

CRISPR-Cas is a bacterial adaptive immune system that was first demonstrated employing a nuclease enzyme (Cas9) that came in 2007 from Barrangou et al. [34]. However, it was Marraffini et al. who proved, in 2008, that CRISPR did not work by RNA interference but by cutting DNA [101]. In parallel, Deveau and Horvath’s groups realized that viral DNA was always digested at the same positions upon infection when the bacterium displayed its CRISPR-Cas immunity system [102,103]. Consequently, they claimed that Cas9 catalyzes the digestion of the DNA at precise positions, encoded by specific sequences of “programmable” RNA (CRISPR-RNA or crRNA), which opened the door to the revolution of CRISPR: a molecular tool that allows accurate site-directed digestion in the DNA. CRISPR can also provide a precise, sensitive diagnostic technique as well as an elegant therapeutic option, which has been applied to identify Zika virus [104], human papillomavirus [105], African Swine Fever virus [106], *Staphylococcus aureus* [107] and *Pseudomona aeruginosa* [108], among others.

### 4.1. CRISPR-Cas as a Molecular Tool of Diagnostic of COVID-19

In the last few months, several projects related to CRISPR have appeared or have been modified in response to the current crisis caused by the COVID-19 pandemic [109]. All these techniques use mainly the Cas13 and Cas12 proteins because of their capacity to cut single-strands of either DNA or RNA [110]. Most of CRISPR based techniques have been developed to use LAMP or RT_LAMP (Reverse transcription loop-mediated isothermal amplification). This technique was developed to simplify the PCR process, with shorter reaction times and no need for specific equipment [111]. Besides, these methods can be developed without high technology or difficulties, allowing the technicians to perform the diagnostic of the disease directly in the sample collection points. Among this research, we highlight six main diagnostic tests using CRISPR technology (Table 2):

(i) SHERLOCK: **S**pecific **H**igh-sensitivity **E**nzymatic **R**eporter un**LOCK**ing. This technique uses the RNAse activity of the CRISPR-Cas13a protein, which needs only a small specific RNA guide [112]. The system was adapted to a simple test against SARS-CoV-2, called STOPCovid (**S**HERLOCK **T**esting in **O**ne **P**ot), which counts nowadays with two versions: STOPCovid.v1 and STOPCovid.v2 [113]. Both of them use LAMP technique for RNA amplification and can detect up to 100 viral genome copies per reaction in 45–60 min. STOPCovid.v2 uses magnetic beads to simplify the RNA extraction and reduce its duration [113]. Researchers have developed a simple test format that can be performed without complex instrumentation and can detect the virus in saliva samples [114]. This method has been clinically validated by a different research group, who have decreased the limit of detection, thus increasing its sensitivity [115].
(ii) DETECTR: **D**NA **E**ndonuclease **T**arg**E**ted **C**RISPR **T**rans **R**eporter. This system uses the CRISPR-Cas12a protein to detect SARS-CoV-2 through its nucleoprotein and envelope genes, based on the method of RT-LAMP, which includes a simultaneous retrotranscription process. This technique allows the detection of the virus in naso- and oropharyngeal samples within 30–40 min. The limit of detection is 10 copies per microliter [116].
(iii) CARMEN: **C**ombinatorial **A**rrayed **R**eactions for **M**ultiplexed **E**valuation of **N**ucleic-acids. This method combines SHERLOCK with microfluidic technology, enabling the analysis of numerous types of samples from patients. The system was developed to detect 169 human-associated viruses, including SARS-CoV-2. Moreover, it can be used for viral detection in several types of samples, ranging from plasma to nasal swab samples [117].
(iv) AIOD-CRISPR: **A**ll **I**n **O**ne **D**ual CRISPR-Cas12a. This system uses the Cas12a protein in a fast, specific, simple method for the visual detection of SARS-CoV-2 and HIV viruses by the naked eye. This method can also be performed at a single temperature, thus avoiding the need for techniques such as LAMP. It detected 1.3 copies of a plasmid expressing the nucleocapsid protein of SARS-CoV-2, although it has not yet been tested with clinical samples [118].
(v) CONAN: **C**as3-**O**perated **N**ucleic **A**cid detectio**N**. This CRISPR-based tool employs mainly Cas3 endonuclease, in combination with Cas5, 6, 7, 8, and 11, which mediates targeted DNA cleavage. When combined with isothermal amplification methods, CONAN provides a rapid and sensitive method to detect SARS-CoV-2, with a reliability of 90% [119].
(vi) CRISPR-COVID: A few months ago, another CRISPR-based tool suitable for the diagnostic of SARS-CoV-2 infection was developed, also based on the Cas13a endonuclease. Scientists claimed that this technique was extremely sensitive and specific, with almost a single-copy sensitivity, as they were able to identify as low as 7.5 copies of viral RNA per reaction in some cases. Furthermore, they did not detect any false positives and the time needed per reaction was only 40 min [120].

Recently, Fozouni and collaborators developed an innovative technique based on the direct detection of SARS-CoV-2 from nasal swab RNA extracts using an amplification-free CRISPR-Cas13a-based mobile phone assay. The sensitivity of the technique was around 100 copies/µL and the duration under 30 min, being able to detect a set of positive clinical samples in under 5 min [121].

### 4.2. CRISPR-Cas as a Treatment

CRISPR technology has also been proposed as a treatment for IAV and COVID-19 by using the PAC-MAN method (**P**rophylactic **A**ntiviral **C**RISPR in hu**MAN** cells). This system uses the Cas13d protein, which has RNAse activity, to destroy the highly conserved genomic RNA regions of the coronavirus. Cas13d enzyme effectively inhibited and degraded SARS-CoV-2 viral RNA in respiratory epithelial cells. The authors suggest several possible delivery forms for the Cas13d protein and its RNA guides, such as nanoparticles, a DNA-based liposomal strategy and a ribonucleoprotein complex. Furthermore, Cas13d is capable of processing its RNA guides so that multiple RNAs with different targets can be delivered at the same time, thus increasing the chances of complete viral eradication. Although this approach has produced promising results in the laboratory, it is still at the pre-clinical trial stage and must be tested in animal models before being tested in humans [122].

Other authors have suggested AAV as a suitable delivery vehicle for the Cas13d, as each viral particle can pack more than three RNA guides. Moreover, AAVs are excellent, safe delivery systems, and they also have specific lung cell serotypes, enabling administration via the respiratory route. Nevertheless, this delivery method, like the treatment based on CRISPR-Cas13, is still at the pre-clinical trial stage [123].

## 5. Discussion

Among this review, we have revised all the viral-based vaccines and viral-related techniques (bacteriophages and CRISPR) that are currently been used in the diagnostic and treatment of SARS-CoV-2. Here, we have summarized all the vaccines that are currently under study (according to WHO), which use viruses as vectors. Moreover, we described the use of phage-display libraries to select monoclonal antibodies specifically against SARS-CoV-2 and how human viruses are used as vectors in vaccines. Finally, in addition to the present techniques, we have reported the new tools that have been developed as new CRISPR diagnosis and treatment methods.

The use of virus-based vaccines has been studied for many years, although until now only one viral-vector vaccine has been approved for use in humans, the rVSV-ZEBOV-GP indicated against Ebola [124]. Nevertheless, some of them are in the final steps of the clinical trials [125]. Despite all the advantages that this kind of vaccines have, they still have some disadvantages such as the pre-existing immunity that can be found against the most common viral-vectors (poxvirus and adenovirus), which might decrease the efficacy of the vaccine [126], or the lack of proper animal models [127]. Nevertheless, in the last months, new murine models have been developed by adding the human ACE-2 receptor to mice by knock-in [128] or transducing the mouse using an adenovirus that expressed the hACE-2 [129]. The viral-vector vaccines are an adequate option in the fight against the COVID-19 disease, being five of the twenty-seven candidate vaccines in a clinical trial to date [73].

However, prophylaxis is not the only way to defeat disease, it is as important to have reliable diagnostic methods and proper treatments. Here we described several new specific diagnostic methods based both in the use of phage-display libraries and CRISPR-Cas. The phage-display libraries are one of the most effective ways to generate a great number of peptides, proteins, or antibodies in a small period [90]. They have been proved useful in the analysis of several autoimmune diseases [130,131] and to produce human antibody therapeutics [132], such as *Helicobacter pylori* [133], *P. aeruginosa* [134], *S. aureus* [135], *Leishmania* [136], Citomegalovirus [137] and Rabies virus [138], among others. However, this technology has a few limitations, like the diversity of the peptides and their quality, which depends on the origin and diversity of the library, as well as on the process employed to evaluate the antibodies [139].

Most of the tools exposed in this review are still under analysis or waiting for their approval, except for the CRISPR-Cas diagnostic systems, some of which are currently accepted with clinical validation. These tools have solved most of the problems that this diagnostic technology had, such as the need for PAM (Protospacer adjacent motif) sequences, quantification of the sample, need to pre-treat the sample or the detection of more than one target per reaction [140]. Interestingly, Fozouni et al. used crRNAs targeting SARS-CoV-2 RNA quantifying viral load using enzyme kinetics, which allows for improvements in the sensitivity and specificity of the diagnosis of COVID19. This innovative assay in combination with mobile phone-based quantification can provide rapid, low-cost, point-of-care screening to aid in the control of SARS-CoV-2 [121]. However, the use of CRISPR technology to treat the disease has to face the main problem of the delivery of the system to the target cells, being proposed several options as phagemids [141] or viruses [142]. Moreover, another problem is the presence of undesirable secondary mutations: although the CRISPR system has a very low frequency of secondary mutations [143], some studies have demonstrated the unnecessary perfect match for the function of the system [144,145].

## 6. Conclusions

This work reviews the ultimate tools already developed and in process for the diagnosis and treatment of the new disease COVID-19 using human viruses, bacteriophages, and the bacterial immune system CRISPR-Cas. These methods are the next step in the development of more specific and precise diagnostic tools as well as a new point of view in the treatment of this pandemic, but also useful for many other diseases. Despite the rapid outcome of all the studies presented here, their results leave no doubt about their usefulness against the SARS-CoV-2. They represent an extraordinary opportunity to defeat this disease as well as an incredible example of a common effort of the scientific community all around the world.

## Figures and Tables

**Table 1:** Viral-vector vaccine candidates and their current state of development according to the WHO.

Developer Institution	Country/s	Type of Viral-Vector	Current State
University of Oxford/ AstraZeneca	United Kingdom	ChAdOx1-S	Clinical trial Phase 3
Beijing Institute of Biotechnology/ CanSino Biological Inc.	China	Ad5	Clinical trial Phase 2
Janssen Pharmaceutical Companies	Belgium	Ad26	Clinical trial Phase ½
Gamaleya Research Institute	Russia	Adenovirus	Clinical trial Phase 1
ReiThera/LEUKOCARE/Uncercells	Italy/Germany/Belgium	Adenovirus	Clinical trial Phase 1
Institute Pasteur/Themis/Univ. of Pittsburgh CVR/Merck Sharp & Dohme	France/United States	Measles	Clinical trial Phase 1
Medicago Inc.	Canada	Plant-derivated VLP	Clinical trial Phase 1
ID Pharma	Japan	Sendai virus	Pre-clinical
Ankara University	Turkey	Adenovirus	Pre-clinical
Massachusetts General Hospital/Massachusetts Eye and Ear/AveXis	United States	Adenovirus	Pre-clinical
GeoVax/BravoVax	United States/China	MVA	Pre-clinical
German center for infection Research/IDT Biologike GmbH	Germany	MVA	Pre-clinical
IDIBAPS-Hospital clinic	Spain	MVA	Pre-clinical
Altimmune	United States	Adenovirus	Pre-clinical
Erciyes University	Turkey	Ad5	Pre-clinical
ImmunityBio Inc/NantKwest Inc.	United States	Ad5	Pre-clinical
Greffex	United States	Ad5	Pre-clinical
Stabilitech Biopharma Ltd.	United Kingdom	Ad5	Pre-clinical
Valo Therapeutics Ltd.	United Kingdom	Adenovirus	Pre-clinical
Vaxart	United States	Ad5	Pre-clinical
National Biotechnology Center (CNB-CSIC)	Spain	MVA	Pre-clinical
University of Georgia/ University of Iowa	United States	Parainfluenza virus	Pre-clinical
Bharat Biotech/Thomas Jefferson University	India/United States	Rabies virus	Pre-clinical
National Research Centre	Egypt	Influenza A	Pre-clinical
National Center for Genetic Engineering and Biotechnology (BIOTEC)/ GPO	Thailand	Flu virus	Pre-clinical
KU Leuven	Belgium	YF17D	Pre-clinical
Cadila Healthcare Limited	India	Measles	Pre-clinical
FBRI SRC VB Vector/ Rospotrebnadzor	Russia	Measles	Pre-clinical
German center for infection Research/ CanVirex AG	Germany	Measles	Pre-clinical
Tonix Pharma/ Southern Research	United States	Horsepox	Pre-clinical
BiOCAD/ IEM	Russia	Influenza	Pre-clinical
FBRI SRC VB Vector/ Rospotrebnadzor	Russia	Influenza A	Pre-clinical
Fundação Oswaldo Cruz/ Instituto Buntantan	Brazil	Influenza	Pre-clinical
University of Hong Kong	China	Influenza	Pre-clinical
IAVI/ Merk	Italy/United States	VSV	Pre-clinical
University of Manitoba	Canada	VSV	Pre-clinical
University of Western Ontario	United States	VSV	Pre-clinical
Aurobindo Pharma	India	VSV	Pre-clinical
FBRI SRC VB Vector/ Rospotrebnadzor	Russia	VSV	Pre-clinical
Israel Institute for Biological Research/ Weizman Institute of Science	Israel	VSV	Pre-clinical
UW-Madison/FluGen/Bharat Biotech	United States	Influenza	Pre-clinical
Intravacc/Wageningen Bioveterinary Research/Utrecht University	The Netherlands	Newcastle disease virus	Pre-clinical
The Lancaster University	United Kingdom	Avian paramyxovirus	Pre-clinical
University of Manitoba	Canada	VLP	Pre-clinical
Bezmialem Vakif University	Turkey	VLP	Pre-clinical
Middle East Technical University	Turkey	VLP	Pre-clinical
VBI Vaccines Inc.	United States	VLP	Pre-clinical
IrsiCaixa AIDS Research/IRTA-CReSA/Barcelona Supercomputing Centre/Grifols	Spain	VLP	Pre-clinical
Mahidol University/The Government Pharmaceutical Organization (GPO)/Siriraj Hospital	Thailand	VLP	Pre-clinical
Navarrabiomed, Oncoinmunology group	Spain	VLP	Pre-clinical
Saiba GmbH	Switzerland	VLP	Pre-clinical
Imophoron Ltd. and Bristol University’s Max Planck Centre	United Kingdom	VLP	Pre-clinical
Doherty Institute	Australia	VLP	Pre-clinical
OSIVAX	France	VLP	Pre-clinical
ARTES Biotechnology	Germany	VLP	Pre-clinical
University of Sao Paulo	Brazil	VLP	Pre-clinical

*VLP—Virus-like particle; VSV—vesicular stomatitis virus.

**Table 2:** Comparison of the main characteristics of some novel diagnostic methods for SARS-CoV-2 and the gold standard COVID-19 RT-PCR assay.

	**COVID-19** **RT-PCR**	**STOP-Covid ^a^ (SHERLOCK)**	**DETECTR**	**CARMEN**	**AIOD-CRISPR**	**CONAN**	**CRISPR-COVID**
**Gene Target**	Spike protein RdRp Nucleocapsid	Spike ORF1ab Nucleocapsid	Envelop Nucleocapsid	ORF1ab	Nucleocapsid	Nucleocapsid	ORF1ab Nucleocapsid
**Sample type**	RNA	RNA	DNA	RNA	DNA	DNA	RNA
**Assay reaction time**	120 min	60 min	30–40 min	~30 min	40 min	30–40 min	40 min
**Nº of samples/ reaction**	1	1	1	1000	1	1	1
**Results**	Quantitative	Semi-quantitative	Qualitative	Quantitative	Quantitative	Quantitative	Qualitative
**Detection limit**	>10 viral copies	42 viral copies	10 viral copies	10 viral copies	1.3 copies of SARS-CoV-2 Nucleocapsid gene plasmids	100 viral copies	7.5 viral copies
**FDA Approval**	Yes	Yes	In process	In process	-	-	-

^a^ This section includes both versions, STOPCovid.v1 and STOPCovid.v2. -: without information.

## References

[1] Drosten C., Günther S., Preiser W., van der Werf S., Brodt H.R., Becker S., Rabenau H., Panning M., Kolesnikova L., Fouchier R.A. (2003). Identification of a novel coronavirus in patients with severe acute respiratory syndrome. N. Engl. J. Med..

[2] Ksiazek T.G., Erdman D., Goldsmith C.S., Zaki S.R., Peret T., Emery S., Tong S., Urbani C., Comer J.A., Lim W. (2003). A novel coronavirus associated with severe acute respiratory syndrome. N. Engl. J. Med..

[3] Cui J., Li F., Shi Z.L. (2019). Origin and evolution of pathogenic coronaviruses. Nat. Rev. Microbiol..

[4] Zhou P., Yang X.L., Wang X.G., Hu B., Zhang L., Zhang W., Si H.R., Zhu Y., Li B., Huang C.L. (2020). A pneumonia outbreak associated with a new coronavirus of probable bat origin. Nature.

[5] Khan M.N., Sarker M. (2020). A review of Coronavirus 2019 COVID-19 a life threating disease all over the world. World Cancer Res. J..

[6] Ahn D.G., Shin H.J., Kim M.H., Lee S., Kim H.S., Myoung J., Kim B.T., Kim S.J. (2020). Current Status of Epidemiology, Diagnosis, Therapeutics, and Vaccines for Novel Coronavirus Disease 2019 (COVID-19). J. Microbiol. Biotechnol..

[7] Wang C., Horby P.W., Hayden F.G., Gao G.F. (2020). A novel coronavirus outbreak of global health concern. Lancet.

[8] World Health Organization (2020). Laboratory testing of 2019 novel coronavirus (2019-nCoV) in suspected human cases. Interim Guidance.

[9] Lisboa Bastos M., Tavaziva G., Abidi S.K., Campbell J.R., Haraoui L.P., Johnston J.C., Lan Z., Law S., MacLean E., Trajman A. (2020). Diagnostic accuracy of serological tests for covid-19: Systematic review and meta-analysis. BMJ.

[10] De Clercq E. (2019). New Nucleoside Analogues for the Treatment of Hemorrhagic Fever Virus Infections. Chem. Asian J..

[11] Wang M., Cao R., Zhang L., Yang X., Liu J., Xu M., Shi Z., Hu Z., Zhong W., Xiao G. (2020). Remdesivir and chloroquine effectively inhibit the recently emerged novel coronavirus (2019-nCoV) in vitro. Cell Res..

[12] Zumla A., Chan J.F., Azhar E.I., Hui D.S., Yuen K.Y. (2016). Coronaviruses—Drug discovery and therapeutic options. Nat. Rev. Drug Discov..

[13] Sheahan T.P., Sims A.C., Graham R.L., Menachery V.D., Gralinski L.E., Case J.B., Leist S.R., Pyrc K., Feng J.Y., Trantcheva I. (2017). Broad-spectrum antiviral GS-5734 inhibits both epidemic and zoonotic coronaviruses. Sci. Transl. Med..

[14] Costanzo M., De Giglio M.A.R., Roviello G.N. (2020). SARS-CoV-2: Recent Reports on Antiviral Therapies Based on Lopinavir/Ritonavir, Darunavir/Umifenovir, Hydroxychloroquine, Remdesivir, Favipiravir and Other Drugs for the Treatment of the New Coronavirus. Curr. Med. Chem..

[15] Neely M., Kalyesubula I., Bagenda D., Myers C., Olness K. (2003). Effect of chloroquine on human immunodeficiency virus (HIV) vertical transmission. Afr. Health Sci..

[16] Vincent M.J., Bergeron E., Benjannet S., Erickson B.R., Rollin P.E., Ksiazek T.G., Seidah N.G., Nichol S.T. (2005). Chloroquine is a potent inhibitor of SARS coronavirus infection and spread. Virol. J..

[17] Freiberg A.N., Worthy M.N., Lee B., Holbrook M.R. (2010). Combined chloroquine and ribavirin treatment does not prevent death in a hamster model of Nipah and Hendra virus infection. J. Gen. Virol..

[18] Dowall S.D., Bosworth A., Watson R., Bewley K., Taylor I., Rayner E., Hunter L., Pearson G., Easterbrook L., Pitman J. (2015). Chloroquine inhibited Ebola virus replication in vitro but failed to protect against infection and disease in the in vivo guinea pig model. J. Gen. Virol..

[19] Chu C.M., Cheng V.C., Hung I.F., Wong M.M., Chan K.H., Chan K.S., Kao R.Y., Poon L.L., Wong C.L., Guan Y. (2004). Role of lopinavir/ritonavir in the treatment of SARS: Initial virological and clinical findings. Thorax.

[20] Boriskin Y.S., Leneva I.A., Pecheur E.I., Polyak S.J. (2008). Arbidol: A broad-spectrum antiviral compound that blocks viral fusion. Curr. Med. Chem..

[21] Duan K., Liu B., Li C., Zhang H., Yu T., Qu J., Zhou M., Chen L., Meng S., Hu Y. (2020). Effectiveness of convalescent plasma therapy in severe COVID-19 patients. Proc. Natl. Acad. Sci. USA.

[22] Ura T., Okuda K., Shimada M. (2014). Developments in Viral Vector-Based Vaccines. Vaccines.

[23] Chen Y.H., Keiser M.S., Davidson B.L. (2018). Viral Vectors for Gene Transfer. Curr. Protoc. Mouse Biol..

[24] Garretto A., Miller-Ensminger T., Wolfe A.J., Putonti C. (2019). Bacteriophages of the lower urinary tract. Nat. Rev. Urol..

[25] Twort F.W. (1915). An investigation on the nature of ultra-microscopic viruses. Lancet.

[26] Furfaro L.L., Payne M.S., Chang B.J. (2018). Bacteriophage Therapy: Clinical Trials and Regulatory Hurdles. Front. Cell Infect. Microbiol..

[27] Schooley R.T., Biswas B., Gill J.J., Hernandez-Morales A., Lancaster J., Lessor L., Barr J.J., Reed S.L., Rohwer F., Benler S. (2017). Development and use of Personalized Bacteriophage-Based Therapeutic Cocktails to Treat a Patient with a Disseminated Resistant Acinetobacter baumannii Infection. Antimicrob. Agents Chemother..

[28] Blasco L., Ambroa A., Trastoy R., Bleriot I., Moscoso M., Fernández-Garcia L., Perez-Nadales E., Fernández-Cuenca F., Torre-Cisneros J., Oteo-Iglesias J. (2020). In vitro and in vivo efficacy of combinations of colistin and different endolysins against clinical strains of multi-drug resistant pathogens. Sci. Rep..

[29] Pacios O., Blasco L., Bleriot I., Fernandez-Garcia L., Gonzalez Bardanca M., Ambroa A., Lopez M., Bou G., Tomas M. (2020). Strategies to Combat Multidrug-Resistant and Persistent Infectious Diseases. Antibiotics.

[30] Goulart L.R., da S.R.V., Costa-Cruz J.M. (2017). Anti-parasitic Antibodies from Phage Display. Adv. Exp. Med. Biol..

[31] Barbas C.F., Burton D.R. (1996). Selection and evolution of high-affinity human anti-viral antibodies. Trends Biotechnol..

[32] Mojica F.J., Juez G., Rodriguez-Valera F. (1993). Transcription at different salinities of Haloferax mediterranei sequences adjacent to partially modified PstI sites. Mol. Microbiol..

[33] Mojica F.J., Diez-Villasenor C., Garcia-Martinez J., Soria E. (2005). Intervening sequences of regularly spaced prokaryotic repeats derive from foreign genetic elements. J. Mol. Evol..

[34] Barrangou R., Fremaux C., Deveau H., Richards M., Boyaval P., Moineau S., Romero D.A., Horvath P. (2007). CRISPR provides acquired resistance against viruses in prokaryotes. Science.

[35] Barrangou R., Marraffini L.A. (2014). CRISPR-Cas systems: Prokaryotes upgrade to adaptive immunity. Mol. Cell.

[36] Lander E.S. (2016). The Heroes of CRISPR. Cell.

[37] Cavazzana-Calvo M., Hacein-Bey S., de Saint Basile G., Gross F., Yvon E., Nusbaum P., Selz F., Hue C., Certain S., Casanova J.L. (2000). Gene therapy of human severe combined immunodeficiency (SCID)-X1 disease. Science.

[38] Tebas P., Stein D., Binder-Scholl G., Mukherjee R., Brady T., Rebello T., Humeau L., Kalos M., Papasavvas E., Montaner L.J. (2013). Antiviral effects of autologous CD4 T cells genetically modified with a conditionally replicating lentiviral vector expressing long antisense to HIV. Blood.

[39] Slobod K.S., Shenep J.L., Lujan-Zilbermann J., Allison K., Brown B., Scroggs R.A., Portner A., Coleclough C., Hurwitz J.L. (2004). Safety and immunogenicity of intranasal murine parainfluenza virus type 1 (Sendai virus) in healthy human adults. Vaccine.

[40] Hansen S.G., Ford J.C., Lewis M.S., Ventura A.B., Hughes C.M., Coyne-Johnson L., Whizin N., Oswald K., Shoemaker R., Swanson T. (2011). Profound early control of highly pathogenic SIV by an effector memory T-cell vaccine. Nature.

[41] Rerks-Ngarm S., Pitisuttithum P., Nitayaphan S., Kaewkungwal J., Chiu J., Paris R., Premsri N., Namwat C., de Souza M., Adams E. (2009). Vaccination with ALVAC and AIDSVAX to prevent HIV-1 infection in Thailand. N. Engl. J. Med..

[42] Coughlan L., Bradshaw A.C., Parker A.L., Robinson H., White K., Custers J., Goudsmit J., Van Roijen N., Barouch D.H., Nicklin S.A. (2012). Ad5:Ad48 hexon hypervariable region substitutions lead to toxicity and increased inflammatory responses following intravenous delivery. Mol. Ther..

[43] Ferreira V., Petry H., Salmon F. (2014). Immune Responses to AAV-Vectors, the Glybera Example from Bench to Bedside. Front. Immunol..

[44] Xin K.Q., Mizukami H., Urabe M., Toda Y., Shinoda K., Yoshida A., Oomura K., Kojima Y., Ichino M., Klinman D. (2006). Induction of robust immune responses against human immunodeficiency virus is supported by the inherent tropism of adeno-associated virus type 5 for dendritic cells. J. Virol..

[45] Perreau M., Pantaleo G., Kremer E.J. (2008). Activation of a dendritic cell-T cell axis by Ad5 immune complexes creates an improved environment for replication of HIV in T cells. J. Exp. Med..

[46] Gomez C.E., Najera J.L., Perdiguero B., Garcia-Arriaza J., Sorzano C.O., Jimenez V., Gonzalez-Sanz R., Jimenez J.L., Munoz-Fernandez M.A., Lopez Bernaldo de Quiros J.C. (2011). The HIV/AIDS vaccine candidate MVA-B administered as a single immunogen in humans triggers robust, polyfunctional, and selective effector memory T cell responses to HIV-1 antigens. J. Virol..

[47] Chiuppesi F., Vannucci L., De Luca A., Lai M., Matteoli B., Freer G., Manservigi R., Ceccherini-Nelli L., Maggi F., Bendinelli M. (2012). A lentiviral vector-based, herpes simplex virus 1 (HSV-1) glycoprotein B vaccine affords cross-protection against HSV-1 and HSV-2 genital infections. J. Virol..

[48] Hansen S.G., Sacha J.B., Hughes C.M., Ford J.C., Burwitz B.J., Scholz I., Gilbride R.M., Lewis M.S., Gilliam A.N., Ventura A.B. (2013). Cytomegalovirus vectors violate CD8 + T cell epitope recognition paradigms. Science.

[49] Cavenaugh J.S., Awi D., Mendy M., Hill A.V., Whittle H., McConkey S.J. (2011). Partially randomized, non-blinded trial of DNA and MVA therapeutic vaccines based on hepatitis B virus surface protein for chronic HBV infection. PLoS ONE.

[50] Tameris M.D., Hatherill M., Landry B.S., Scriba T.J., Snowden M.A., Lockhart S., Shea J.E., McClain J.B., Hussey G.D., Hanekom W.A. (2013). Safety and efficacy of MVA85A, a new tuberculosis vaccine, in infants previously vaccinated with BCG: A randomised, placebo-controlled phase 2b trial. Lancet.

[51] Smaill F., Jeyanathan M., Smieja M., Medina M.F., Thanthrige-Don N., Zganiacz A., Yin C., Heriazon A., Damjanovic D., Puri L. (2013). A human type 5 adenovirus-based tuberculosis vaccine induces robust T cell responses in humans despite preexisting anti-adenovirus immunity. Sci. Transl. Med..

[52] Lin J., Calcedo R., Vandenberghe L.H., Bell P., Somanathan S., Wilson J.M. (2009). A new genetic vaccine platform based on an adeno-associated virus isolated from a rhesus macaque. J. Virol..

[53] Berthoud T.K., Hamill M., Lillie P.J., Hwenda L., Collins K.A., Ewer K.J., Milicic A., Poyntz H.C., Lambe T., Fletcher H.A. (2011). Potent CD8 + T-cell immunogenicity in humans of a novel heterosubtypic influenza A vaccine, MVA-NP+M1. Clin. Infect. Dis..

[54] Carter B.J. (2005). Adeno-associated virus vectors in clinical trials. Hum. Gene Ther..

[55] Chan W.M., Rahman M.M., McFadden G. (2013). Oncolytic myxoma virus: The path to clinic. Vaccine.

[56] Jiang S., Hillyer C., Du L. (2020). Neutralizing Antibodies against SARS-CoV-2 and Other Human Coronaviruses. Trends Immunol..

[57] Chiuppesi F., Salazar M.D., Contreras H., Nguyen V.H., Martinez J., Park S., Nguyen J., Kha M., Iniguez A., Zhou Q. (2020). Development of a Synthetic Poxvirus-Based SARS-CoV-2 Vaccine. bioRxiv.

[58] Wold W.S., Toth K. (2013). Adenovirus vectors for gene therapy, vaccination and cancer gene therapy. Curr. Gene.

[59] Zhu F.C., Guan X.H., Li Y.H., Huang J.Y., Jiang T., Hou L.H., Li J.X., Yang B.F., Wang L., Wang W.J. (2020). Immunogenicity and safety of a recombinant adenovirus type-5-vectored COVID-19 vaccine in healthy adults aged 18 years or older: A randomised, double-blind, placebo-controlled, phase 2 trial. Lancet.

[60] US National Library of Medicine (2020). Phase III Trial of A COVID-19 Vaccine of Adenovirus Vector in Adults 18 Years Old and above, on NIH.

[61] Van Doremalen N., Lambe T., Spencer A., Belij-Rammerstorfer S., Purushotham J.N., Port J.R., Avanzato V.A., Bushmaker T., Flaxman A., Ulaszewska M. (2020). ChAdOx1 nCoV-19 vaccination prevents SARS-CoV-2 pneumonia in rhesus macaques. Nature.

[62] Folegatti P.M., Ewer K.J., Aley P.K., Angus B., Becker S., Belij-Rammerstorfer S., Bellamy D., Bibi S., Bittaye M., Clutterbuck E.A. (2020). Safety and immunogenicity of the ChAdOx1 nCoV-19 vaccine against SARS-CoV-2: A preliminary report of a phase 1/2, single-blind, randomised controlled trial. Lancet.

[63] US National Library of Medicine (2020). Phase III Doubled-Blind, Placebo-Controlled Study of AZD1222 for the Prevention of COVID-19 in Adults, on NIH.

[64] Mercado N.B., Zahn R., Wegmann F., Loos C., Chandrashekar A., Yu J., Liu J., Peter L., McMahan K., Tostanoski L.H. (2020). Single-shot Ad26 vaccine protects against SARS-CoV-2 in rhesus macaques. Nature.

[65] Janssen Pharmaceutical Companies A Study of Ad26COVS1 in Adults (COVID-19). https://clinicaltrials.gov/ct2/show/record/NCT04436276?term=NCT04436276&draw=2&rank=1.

[66] Companies J.P. (2020). A Study of Ad26.COV2.S for the Prevention of SARS-CoV-2-Mediated COVID-19 in Adult Participants (ENSEMBLE), on NIH.

[67] Gamaleya Research Institute An Open Study of the Safety, Tolerability and Immunogenicity of the Drug “Gam-COVID-Vac” Vaccine Against COVID-19. https://clinicaltrials.gov/ct2/show/NCT04436471?term=vaccine&cond=covid-19&draw=4.

[68] Gamaleya Research Institute An Open Study of the Safety, Tolerability and Immunogenicity of “Gam-COVID-Vac Lyo” Vaccine Against COVID-19. https://clinicaltrials.gov/ct2/show/record/NCT04437875?term=vaccine&cond=covid-19&draw=4.

[69] ReiThera European Consortium for the Fast-Track Development of a Single-Dose Adenovirus-Based COVID-19 Vaccine. https://www.reithera.com/2020/04/23/reithera-leukocare-and-univercells-announce-pan-european-consortium-for-the-fast-track-development-of-a-single-dose-adenovirus-based-covid-19-vaccine/.

[70] Institute Pasteur Clinical Trial to Evaluate the Safety and Immunogenicitiy of the COVID-19 Vaccine (COVID-19-101). https://clinicaltrials.gov/ct2/show/record/NCT04497298?term=vaccine&cond=covid-19&draw=2&rank=1.

[71] Medicago Inc Safety, Tolerability and Immunogenicinity of a Coronavirus-Like Particle COVID-19 Vaccine in Adults Aged 18–55 Years. https://clinicaltrials.gov/ct2/show/record/NCT04450004?term=vaccine&cond=covid-19&draw=2.

[72] University Xiamen (2020). A Phase I Clinical Trial of Influenza Virus Vector COVID-19 Vaccine for Intranasal Spray.

[73] World Health Organization Draft Landscape of COVID-19 Candidate Vaccines. 9 September 2020. https://www.who.int/publications/m/item/draft-landscape-of-covid-19-candidate-vaccines.

[74] De la Cruz V.F., Lal A.A., McCutchan T.F. (1988). Immunogenicity and epitope mapping of foreign sequences via genetically engineered filamentous phage. J. Biol. Chem..

[75] Wang C., Sun X., Suo S., Ren Y., Li X., Herrler G., Thiel V., Ren X. (2013). Phages bearing affinity peptides to severe acute respiratory syndromes-associated coronavirus differentiate this virus from other viruses. J. Clin. Virol..

[76] Ebrahimizadeh W., Rajabibazl M. (2014). Bacteriophage vehicles for phage display: Biology, mechanism, and application. Curr. Microbiol..

[77] Marks J.D., Hoogenboom H.R., Griffiths A.D., Winter G. (1992). Molecular evolution of proteins on filamentous phage. Mimicking the strategy of the immune system. J. Biol. Chem..

[78] Christensen D.J., Gottlin E.B., Benson R.E., Hamilton P.T. (2001). Phage display for target-based antibacterial drug discovery. Drug Discov. Today.

[79] Kretzschmar T., von Ruden T. (2002). Antibody discovery: Phage display. Curr. Opin. Biotechnol..

[80] Hong S.S., Boulanger P. (1995). Protein ligands of the human adenovirus type 2 outer capsid identified by biopanning of a phage-displayed peptide library on separate domains of wild-type and mutant penton capsomers. EMBO J..

[81] Dyson M.R., Murray K. (1995). Selection of peptide inhibitors of interactions involved in complex protein assemblies: Association of the core and surface antigens of hepatitis B virus. Proc. Natl. Acad. Sci. USA.

[82] Larson R.S., Brown D.C., Ye C., Hjelle B. (2005). Peptide antagonists that inhibit Sin Nombre virus and hantaan virus entry through the beta3-integrin receptor. J. Virol..

[83] Hall P.R., Hjelle B., Njus H., Ye C., Bondu-Hawkins V., Brown D.C., Kilpatrick K.A., Larson R.S. (2009). Phage display selection of cyclic peptides that inhibit Andes virus infection. J. Virol..

[84] Qi H., Lu H., Qiu H.J., Petrenko V., Liu A. (2012). Phagemid vectors for phage display: Properties, characteristics and construction. J. Mol. Biol..

[85] Marintcheva B. (2018). Chapter 5—Phage Display. Harnessing the Power of Viruses.

[86] Hess G.T., Cragnolini J.J., Popp M.W., Allen M.A., Dougan S.K., Spooner E., Ploegh H.L., Belcher A.M., Guimaraes C.P. (2012). M13 bacteriophage display framework that allows sortase-mediated modification of surface-accessible phage proteins. Bioconjug. Chem..

[87] Wu J., Tu C., Yu X., Zhang M., Zhang N., Zhao M., Nie W., Ren Z. (2007). Bacteriophage T4 nanoparticle capsid surface SOC and HOC bipartite display with enhanced classical swine fever virus immunogenicity: A powerful immunological approach. J. Virol. Methods.

[88] Krumpe L.R., Atkinson A.J., Smythers G.W., Kandel A., Schumacher K.M., McMahon J.B., Makowski L., Mori T. (2006). T7 lytic phage-displayed peptide libraries exhibit less sequence bias than M13 filamentous phage-displayed peptide libraries. Proteomics.

[89] Cicchini C., Ansuini H., Amicone L., Alonzi T., Nicosia A., Cortese R., Tripodi M., Luzzago A. (2002). Searching for DNA-protein interactions by lambda phage display. J. Mol. Biol..

[90] Bazan J., Calkosinski I., Gamian A. (2012). Phage display—A powerful technique for immunotherapy: 1. Introduction and potential of therapeutic applications. Hum. Vaccin. Immunother..

[91] Chakravarthy B., Ménard M., Brown L., Atkinson T., Whitfield J. (2012). Identification of protein kinase C inhibitory activity associated with a polypeptide isolated from a phage display system with homology to PCM-1, the pericentriolar material-1 protein. Biochem. Biophys. Res. Commun..

[92] Liu Z.X., Yi G.H., Qi Y.P., Liu Y.L., Yan J.P., Qian J., Du E.Q., Ling W.F. (2005). Identification of single-chain antibody fragments specific against SARS-associated coronavirus from phage-displayed antibody library. Biochem. Biophys. Res. Commun.

[93] Lim C.C., Woo P.C.Y., Lim T.S. (2019). Development of a Phage Display Panning Strategy Utilizing Crude Antigens: Isolation of MERS-CoV Nucleoprotein human antibodies. Sci. Rep..

[94] Duan J., Yan X., Guo X., Cao W., Han W., Qi C., Feng J., Yang D., Gao G., Jin G. (2005). A human SARS-CoV neutralizing antibody against epitope on S2 protein. Biochem. Biophys. Res. Commun..

[95] Kang X., Yang B.A., Hu Y., Zhao H., Xiong W., Yang Y., Si B., Zhu Q. (2006). Human neutralizing Fab molecules against severe acute respiratory syndrome coronavirus generated by phage display. Clin. Vaccine Immunol..

[96] Begum N., Horiuchi S., Tanaka Y., Yamamoto N., Ichiyama K., Yamamoto N. (2002). New approach for generation of neutralizing antibody against human T-cell leukaemia virus type-I (HTLV-I) using phage clones. Vaccine.

[97] Wu Y., Li C., Xia S., Tian X., Kong Y., Wang Z., Gu C., Zhang R., Tu C., Xie Y. (2020). Identification of Human Single-Domain Antibodies against SARS-CoV-2. Cell Host Microbe.

[98] Zeng X., Li L., Lin J., Li X., Liu B., Kong Y., Zeng S., Du J., Xiao H., Zhang T. (2020). Blocking antibodies against SARS-CoV-2 RBD isolated from a phage display antibody library using a competitive biopanning strategy. bioRxiv.

[99] Ren X., Liu B., Yin J., Zhang H., Li G. (2011). Phage displayed peptides recognizing porcine aminopeptidase N inhibit transmissible gastroenteritis coronavirus infection in vitro. Virology.

[100] Lauster D., Klenk S., Ludwig K., Nojoumi S., Behren S., Adam L., Stadtmüller M., Saenger S., Zimmler S., Hönzke K. (2020). Phage capsid nanoparticles with defined ligand arrangement block influenza virus entry. Nat. Nanotechnol..

[101] Marraffini L.A., Sontheimer E.J. (2008). CRISPR interference limits horizontal gene transfer in staphylococci by targeting DNA. Science.

[102] Deveau H., Barrangou R., Garneau J.E., Labonte J., Fremaux C., Boyaval P., Romero D.A., Horvath P., Moineau S. (2008). Phage response to CRISPR-encoded resistance in Streptococcus thermophilus. J. Bacteriol..

[103] Horvath P., Romero D.A., Coute-Monvoisin A.C., Richards M., Deveau H., Moineau S., Boyaval P., Fremaux C., Barrangou R. (2008). Diversity, activity, and evolution of CRISPR loci in Streptococcus thermophilus. J. Bacteriol..

[104] Pardee K., Green A.A., Takahashi M.K., Braff D., Lambert G., Lee J.W., Ferrante T., Ma D., Donghia N., Fan M. (2016). Rapid, Low-Cost Detection of Zika Virus Using Programmable Biomolecular Components. Cell.

[105] Chen J.S., Ma E., Harrington L.B., Da Costa M., Tian X., Palefsky J.M., Doudna J.A. (2018). CRISPR-Cas12a target binding unleashes indiscriminate single-stranded DNase activity. Science.

[106] He Q., Yu D., Bao M., Korensky G., Chen J., Shin M., Kim J., Park M., Qin P., Du K. (2020). High-throughput and all-solution phase African Swine Fever Virus (ASFV) detection using CRISPR-Cas12a and fluorescence based point-of-care system. Biosens. Bioelectron..

[107] Guk K., Keem J.O., Hwang S.G., Kim H., Kang T., Lim E.K., Jung J. (2017). A facile, rapid and sensitive detection of MRSA using a CRISPR-mediated DNA FISH method, antibody-like dCas9/sgRNA complex. Biosens. Bioelectron..

[108] Gootenberg J.S., Abudayyeh O.O., Kellner M.J., Joung J., Collins J.J., Zhang F. (2018). Multiplexed and portable nucleic acid detection platform with Cas13, Cas12a, and Csm6. Science.

[109] Davies K., Barrangou R. (2020). COVID-19 and the CRISPR Community Response. Cris. J..

[110] Wang X., Shang X., Huang X. (2020). Next-generation pathogen diagnosis with CRISPR/Cas-based detection methods. Emerg. Microbes Infect..

[111] Li J.J., Xiong C., Liu Y., Liang J.S., Zhou X.W. (2016). Loop-Mediated Isothermal Amplification (LAMP): Emergence as an Alternative Technology for Herbal Medicine Identification. Front. Plant Sci..

[112] Kellner M.J., Koob J.G., Gootenberg J.S., Abudayyeh O.O., Zhang F. (2019). SHERLOCK: Nucleic acid detection with CRISPR nucleases. Nat. Protoc..

[113] Joung J., Ladha A., Saito M., Kim N.G., Woolley A.E., Segel M., Barretto R.P.J., Ranu A., Macrae R.K., Faure G. (2020). Detection of SARS-CoV-2 with SHERLOCK One-Pot Testing. N. Engl. J. Med..

[114] Joung J., Ladha A., Saito M., Segel M., Bruneau R., Huang M.-l.W., Kim N.-G., Yu X., Li J., Walker B.D. (2020). Point-of-care testing for COVID-19 using SHERLOCK diagnostics. MedRxiv.

[115] Patchsung M., Jantarug K., Pattama A., Aphicho K., Suraritdechachai S., Meesawat P., Sappakhaw K., Leelahakorn N., Ruenkam T., Wongsatit T. (2020). Clinical validation of a Cas13-based assay for the detection of SARS-CoV-2 RNA. Nat. Biomed. Eng..

[116] Broughton J.P., Deng X., Yu G., Fasching C.L., Servellita V., Singh J., Miao X., Streithorst J.A., Granados A., Sotomayor-Gonzalez A. (2020). CRISPR-Cas12-based detection of SARS-CoV-2. Nat. Biotechnol..

[117] Ackerman C.M., Myhrvold C., Thakku S.G., Freije C.A., Metsky H.C., Yang D.K., Ye S.H., Boehm C.K., Kosoko-Thoroddsen T.-S.F., Kehe J. (2020). Massively multiplexed nucleic acid detection using Cas13. Nature.

[118] Ding X., Yin K., Li Z., Liu C. (2020). All-in-One Dual CRISPR-Cas12a (AIOD-CRISPR) Assay: A Case for Rapid, Ultrasensitive and Visual Detection of Novel Coronavirus SARS-CoV-2 and HIV virus. bioRxiv.

[119] Yoshimi K., Takeshita K., Yamayoshi S., Shibumura S., Yamauchi Y., Yamamoto M., Yotsuyanagi H., Kawaoka Y., Mashimo T. (2020). Rapid and accurate detection of novel coronavirus SARS-CoV-2 using CRISPR-Cas3. MedRxiv.

[120] Hou T., Zeng W., Yang M., Chen W., Ren L., Ai J., Wu J., Liao Y., Gou X., Li Y. (2020). Development and evaluation of a rapid CRISPR-based diagnostic for COVID-19. PLoS Pathog..

[121] Abbott T.R., Dhamdhere G., Liu Y., Lin X., Goudy L., Zeng L., Chemparathy A., Chmura S., Heaton N.S., Debs R. (2020). Development of CRISPR as an Antiviral Strategy to Combat SARS-CoV-2 and Influenza. Cell.

[122] Fozouni P., Son S., de León Derby M.D., Knott G.J., Gray C.N., D’Ambrosio M.V., Zhao C., Switz N.A., Kumar G.R., Stephens S.I. (2020). Direct detection of SARS-CoV-2 using CRISPR-Cas13a and a mobile phone. MedRxiv.

[123] Nguyen T.M., Zhang Y., Pandolfi P.P. (2020). Virus against virus: A potential treatment for 2019-nCov (SARS-CoV-2) and other RNA viruses. Cell Res..

[124] USA Food and Drugs Agency First FDA-Approved Vaccine for the Prevention of Ebola Virus Disease, Marking a Critical Milestone in Public Health Preparedness and Response. https://www.fda.gov/news-events/press-announcements/first-fda-approved-vaccine-prevention-ebola-virus-disease-marking-critical-milestone-public-health.

[125] Coughlan L. (2020). Factors Which Contribute to the Immunogenicity of Non-replicating Adenoviral Vectored Vaccines. Front. Immunol..

[126] Cooney E.L., Collier A.C., Greenberg P.D., Coombs R.W., Zarling J., Arditti D.E., Hoffman M.C., Hu S.L., Corey L. (1991). Safety of and immunological response to a recombinant vaccinia virus vaccine expressing HIV envelope glycoprotein. Lancet.

[127] Lurie N., Saville M., Hatchett R., Halton J. (2020). Developing Covid-19 Vaccines at Pandemic Speed. N. Engl. J. Med..

[128] Sun S.H., Chen Q., Gu H.J., Yang G., Wang Y.X., Huang X.Y., Liu S.S., Zhang N.N., Li X.F., Xiong R. (2020). A Mouse Model of SARS-CoV-2 Infection and Pathogenesis. Cell Host Microbe.

[129] Sun J., Zhuang Z., Zheng J., Li K., Wong R.L., Liu D., Huang J., He J., Zhu A., Zhao J. (2020). Generation of a Broadly Useful Model for COVID-19 Pathogenesis, Vaccination, and Treatment. Cell.

[130] Latrofa F., Pichurin P., Guo J., Rapoport B., McLachlan S.M. (2003). Thyroglobulin-thyroperoxidase autoantibodies are polyreactive, not bispecific: Analysis using human monoclonal autoantibodies. J. Clin. Endocrinol. Metab..

[131] Payne A.S., Ishii K., Kacir S., Lin C., Li H., Hanakawa Y., Tsunoda K., Amagai M., Stanley J.R., Siegel D.L. (2005). Genetic and functional characterization of human pemphigus vulgaris monoclonal autoantibodies isolated by phage display. J. Clin. Investig..

[132] Venkatesh N., Im S.H., Balass M., Fuchs S., Katchalski-Katzir E. (2000). Prevention of passively transferred experimental autoimmune myasthenia gravis by a phage library-derived cyclic peptide. Proc. Natl. Acad. Sci. USA.

[133] Houimel M., Corthesy-Theulaz I., Fisch I., Wong C., Corthesy B., Mach J., Finnern R. (2001). Selection of human single chain Fv antibody fragments binding and inhibiting Helicobacter pylori urease. Tumour. Biol..

[134] Molina-Lopez J., Sanschagrin F., Levesque R.C. (2006). A peptide inhibitor of MurA UDP-N-acetylglucosamine enolpyruvyl transferase: The first committed step in peptidoglycan biosynthesis. Peptides.

[135] Yacoby I., Shamis M., Bar H., Shabat D., Benhar I. (2006). Targeting antibacterial agents by using drug-carrying filamentous bacteriophages. Antimicrob. Agents Chemother..

[136] Coelho E.A., Chavez-Fumagalli M.A., Costa L.E., Tavares C.A., Soto M., Goulart L.R. (2015). Theranostic applications of phage display to control leishmaniasis: Selection of biomarkers for serodiagnostics, vaccination, and immunotherapy. Rev. Soc. Bras. Med. Trop..

[137] Carlsson F., Trilling M., Perez F., Ohlin M. (2012). A dimerized single-chain variable fragment system for the assessment of neutralizing activity of phage display-selected antibody fragments specific for cytomegalovirus. J. Immunol. Methods.

[138] Zhao X.L., Yin J., Chen W.Q., Jiang M., Yang G., Yang Z.H. (2008). Generation and characterization of human monoclonal antibodies to G5, a linear neutralization epitope on glycoprotein of rabies virus, by phage display technology. Microbiol. Immunol..

[139] Rahbarnia L., Farajnia S., Babaei H., Majidi J., Veisi K., Ahmadzadeh V., Akbari B. (2017). Evolution of phage display technology: From discovery to application. J Drug Target.

[140] Li Y., Li S., Wang J., Liu G. (2019). CRISPR/Cas Systems towards Next-Generation Biosensing. Trends Biotechnol..

[141] Citorik R.J., Mimee M., Lu T.K. (2014). Sequence-specific antimicrobials using efficiently delivered RNA-guided nucleases. Nat. Biotechnol..

[142] Xu C.L., Ruan M.Z.C., Mahajan V.B., Tsang S.H. (2019). Viral Delivery Systems for CRISPR. Viruses.

[143] Jiang W., Marraffini L.A. (2015). CRISPR-Cas: New Tools for Genetic Manipulations from Bacterial Immunity Systems. Annu. Rev. Microbiol..

[144] Jackson R.N., Golden S.M., van Erp P.B., Carter J., Westra E.R., Brouns S.J., van der Oost J., Terwilliger T.C., Read R.J., Wiedenheft B. (2014). Structural biology. Crystal structure of the CRISPR RNA-guided surveillance complex from Escherichia coli. Science.

[145] Gasiunas G., Barrangou R., Horvath P., Siksnys V. (2012). Cas9-crRNA ribonucleoprotein complex mediates specific DNA cleavage for adaptive immunity in bacteria. Proc. Natl. Acad. Sci. USA.

